# Influence of bacterial and alveolar cell co-culture on microbial VOC production using HS-GC/MS

**DOI:** 10.3389/fmolb.2023.1160106

**Published:** 2023-04-26

**Authors:** Dominic Fenn, Waqar M. Ahmed, Thijs A. Lilien, Renate Kos, Anita M. Tuip de Boer, Stephen J. Fowler, Marcus J. Schultz, Anke H. Maitland-van der Zee, Paul Brinkman, Lieuwe D. J. Bos

**Affiliations:** ^1^ Department of Pulmonary medicine, Amsterdam UMC, University of Amsterdam, Amsterdam, Netherlands; ^2^ Laboratory of Experimental Intensive Care and Anaesthesiology, Amsterdam UMC, University of Amsterdam, Amsterdam, Netherlands; ^3^ Division of Immunology, Immunity to Infection and Respiratory Medicine, Faculty of Biology, Medicine and Health, University of Manchester, Manchester, United Kingdom; ^4^ NIHR-Manchester Biomedical Research Centre, Manchester University Hospitals NHS Foundation Trust, Amsterdam, United Kingdom; ^5^ Paediatric Intensive Care Unit, Emma Children’s Hospital, Amsterdam UMC, University of Amsterdam, Amsterdam, Netherlands; ^6^ Intensive Care, Amsterdam UMC, University of Amsterdam, Amsterdam, Netherlands

**Keywords:** VOC, HS-GC/MS, culture media composition, co-culture model, bacterial VOCs

## Abstract

Volatile organic compounds (VOCs) found in exhaled breath continue to garner interest as an alternative diagnostic tool in pulmonary infections yet, their clinical integration remains a challenge with difficulties in translating identified biomarkers. Alterations in bacterial metabolism secondary to host nutritional availability may explain this but is often inadequately modelled *in vitro*. The influence of more clinically relevant nutrients on VOC production for two common respiratory pathogens was investigated. VOCs from *Staphylococcus aureus* (*S.aureus*) and *Pseudomonas aeruginosa* (*P.aeruginosa*) cultured with and without human alveolar A549 epithelial cells were analyzed using headspace extraction coupled with gas chromatography-mass spectrometry. Untargeted and targeted analyses were performed, volatile molecules identified from published data, and the differences in VOC production evaluated. Principal component analysis (PCA) could differentiate alveolar cells from either *S. aureus* or *P. aeruginosa* when cultured in isolation based on PC1 (*p* = 0.0017 and 0.0498, respectively). However, this separation was lost for *S. aureus* (*p* = 0.31) but not for *P. aeruginosa* (*p* = 0.028) when they were cultured with alveolar cells*. S. aureus* cultured with alveolar cells led to higher concentrations of two candidate biomarkers, 3-methyl-1-butanol (*p* = 0.001) and 3-methylbutanal (*p* = 0.002) when compared to *S. aureus,* alone. *P. aeruginosa* metabolism resulted in less generation of pathogen-associated VOCs when co-cultured with alveolar cells compared to culturing in isolation. VOC biomarkers previously considered indicative of bacterial presence are influenced by the local nutritional environment and this should be considered when evaluating their biochemical origin.

## Introduction

Pulmonary infections remain the leading cause of communicable deaths worldwide (Hubbard, 2006). Continued efforts are being made to modernise clinical microbiology and improve pathogen detection. However, whilst molecular diagnostics are readily becoming recognised as the new gold standard in clinical virology replacing the need for viral cultures (Hodinka and Kaiser, 2013), the same is not true for clinical microbiology that remains reliant on culture-based methodologies (Didelot et al., 2012; Chanderraj and Dickson, 2018). Consequently, clinical microbiology remains at the mercy of culture dependent analysis and as such, is limited by a) sample availability and b) whether the causative organism can be cultured. Furthermore, conventional cultures are a laborious and inherently biased approach associated with delays in targeted antibiotic therapy and broad-spectrum antibiotic over use (Joo et al., 2014; Hilton et al., 2016). An alternative approach is therefore long overdue.

Exhaled breath metabolomics with its ease of collection and sputum independence represents a technique that could overcome these limitations (Filipiak et al., 2015; Hilton et al., 2016; Kos et al., 2021; Ahmed et al., 2022). In addition, the characterisation and detection of changes in metabolic activity, that may precede disease symptoms, could provide invaluable information regarding bacterial presence, viability and activity (Tounta et al., 2021; Mohd Kamal et al., 2022). However, as with all omics data, the challenge lies in clinically integration (van Karnebeek et al., 2018). Presently, there is a surfeit of discovery exhaled breath studies identifying various volatile organic compounds (VOCs) as candidate biomarkers for specific bacteria (Filipiak et al., 2012; 2015; Fenn et al., 2021). Yet, translational difficulties between the pre-clinically and clinically identified VOCs (Filipiak et al., 2015; Ahmed et al., 2022) prevent their necessary validation and subsequent application.

Successful pathogenic bacterial colonisation is dependent on respiratory tract surface adherence and the procurement of local nutrients for growth (Siegel and Weiser, 2015). Consequently, bacteria are capable of adapting their metabolism in response to an altered nutritional availability at the infection site (Brown et al., 2008; Siegel and Weiser, 2015). The impact of this metabolic alteration is frequently over looked in *vitro* VOC studies and may partly explain the observed translational difficulties. In the present study, two common respiratory pathogens, i.e., *Staphylococcus aureus* (*S.aureus*) and *Pseudomonas aeruginosa* (*P.aeruginosa*) were co-cultured with alveolar epithelial cells, and analyzed using headspace extraction coupled with gas chromatography-mass spectrometry (HS-GC/MS) to explore the impact of the nutritional environment on microbial VOC production. It was hypothesised that bacteria cultured in a growth substrate more representative of *in vivo* conditions would a) yield separate VOCs, and b) the VOCs that have previously been identified *in vivo* would yield higher concentrations in the co-cultured headspace than in regular headspace analysis.

## Materials and methods

### Cell line and bacterial cultivation

For the cellular component, culture conditions and sample preparation were as described previously (Fenn et al., 2022). In brief, immortalised human alveolar basal epithelial (A549) cells (CCL-185) were cultivated in 75 cm^2^ cell culture flasks and incubated at 37 °C in 5% CO2 in Roswell Park Memorial Institute (RPMI) 1,640 medium (Gibco) supplemented with 10% foetal bovine serum (FBS), Penicillin-Streptomycin (5 mL containing 10,000 units per mL Penicillin, 10,000 μg mL^−1^ Streptomycin, Gibco), L-Glutamine, gentamicin and amphotericin B. Cells were passaged every three to 4 days once ≈90% confluent with a similar passage used for each experimental replicate. Prior to headspace collection and bacterial inoculation, they were detached from the culture flask using 0.05% Trypsin-EDTA and resuspended in RPMI-1640 before being seeded (≈1.5 × 10^5^) in 1 mL of supplemented RPMI-1640 in 20 mL glass headspace vials (Markes International, Bridgend, Wales) and incubated at 37°C in 5% CO_2_ for 22–24 h (Fenn et al., 2022).

The reference strains *S. aureus* (ATCC 29213), *P. aeruginosa* (PAO1) were investigated. For each experimental replicate, strains were sub-cultured from glycerol frozen stocks onto Columbia blood agar plates with 5% sheep blood (43,049, bioMérieux, Marcy-l'Étoile, France) and incubated over night at 37°C to ensure axenic colonies. Isolated colonies were then inoculated and cultivated in 20 mL brain heart infusion (BHI, nr: 116, Amsterdam UMC—location AMC, Netherlands) and grown overnight at 37°C without agitation.

### Co-culture infection and treatment experiments

Overnight liquid cultures for both *S. aureus* and *P. aeruginosa* were firstly standardised to a 0.35 OD_620nm_ using RPMI-1640 media without antibiotics. A stepwise dilution was then done to achieve a target of 10^4^ colony forming units per ml (CFU/mL), a frequently used diagnostic threshold to define a positive culture *in vivo* (Baselski and Klutts, 2013).

Once the A549 cells had formed a monolayer with a confluence ≈80–90% within the glass headspace vials, the medium was removed. The vials were then washed with phosphate buffered saline to remove any residual traces of RMPI media containing antibiotics. The cells were then replenished with a) 200 ul antibiotic free RPMI-1640 media (control), b) 200 ul standardised *S. aureus* inoculate or c) 200 ul standardised *P. aeruginosa* inoculate prepared in antibiotic free RPMI-1640 media. For the bacteria cultured without cells, the headspace vials were treated in a similar fashion prior to inoculation, using RPMI media alone instead of A549 cells. 200 ul of the standardised inoculate was then added for each bacterium to the glass headspace vials. All vials were sealed with crimp-tops with polytetrafluoroethylene (PTFE)-lined septa (Markes International, Cincinnati, Ohio, United States) and incubated and agitated for 24 h (temperature 37°C, agitation 200 RPM, HiSorb agitator, Markes International, Cincinnati, Ohio, United States). The experiment was conducted in triplicate with three biological replicates per condition per experimental day. Quantitative culturing was performed to determine the CFU/mL of the standardised inoculates for each experiment. Replicates were removed from analysis if target CFU count was not met. Similarly, sterility assessments for the control group were also performed following headspace capture to ensure no evidence of infection.

### HS-GC/MS

Headspace sampling was performed using HiSorb high-capacity extraction probes with polydimethylsiloxane sorbent phase (Markes International, Cincinnati, Ohio, United States) as previous described (Fenn et al., 2022). In brief, following inoculation the 20 mL glass headspace vials were sealed and incubated and agitated for 24 h. Conditioned sorbent probes were then inserted into the headspace for 2 h after 22 h of incubation. The probes were removed and stored in empty stainless steel sorbent tubes (Markes International, Cincinnati, Ohio, United States) ahead of thermal desorption. HS-GC/MS analysis was performed on the same day as VOC capture for all samples as previously described (Fenn et al., 2022). Compounds of interest were identified using the GC/MS Solutions (Shimadzu, Den Bosch, the Netherlands) platform incorporating the National Institute and Technology library as described previously (Bos et al., 2014) and compared to analytical standard where possible. Any compounds that had match score < 80 or could not be accurately detected due to co-elution were excluded from further analysis.

### Statistical analysis and data processing

Statistical analysis was performed through the R studio interface using R (version 3.6.1). Raw HS-GC/MS spectra were processed using the R “xcms’ package (Scripps Center for Metabolomics, La Jolla, CA, United States) as previously reported (Bos et al., 2014), and underwent denoising, peak detection and alignment to create a three-dimensional data matrix containing sample metadata, retention time and mass-to-charge ratio (m/z), ahead of downstream analysis. Failed analyses and technical errors were excluded by visual inspection of chromatograms after processing using the “xcms” pipeline. Known analytical artefacts, such as siloxanes were also removed. The peak table was then normalised using “limma” package and log scaled to adjust for experimental day differences and to stabilise variance.

An untargeted and targeted approach were used to explore VOC differences in bacteria cultured with and without A549 cells. In the untargeted approach, variation of VOC profiles between bacteria and A549 cells in isolation was assessed using principal component analysis (PCA). Loadings from this PCA were projected on samples from bacteria cultured with cells and differences between group centroids were assessed using pairwise *post hoc* Dunn’s analysis correcting for multiple testing. VOC concentrations were then compared between bacteria and bacteria cultured with cells using the Mann-Whitney *U* test, and evaluated alongside a log2 fold change. This was visualised using a volcano plot. A log2 fold change ≥2 and an *p*-value <0.05 was selected to limit false discovery and ensure biologically meaningful differential VOC expression. The analyses were split for each pathogen.

For the targeted approach, species-specific volatile metabolites were identified from previously conducted systematic literature reviews (Kos et al., 2021; Kos et al., unpublished data) and further filtered to include only VOCs that have been reported in one or more *in vivo* studies (Supplementary Table S1). Mann-Whitney U analysis was used to compare VOC concentrations for each pathogen, cultured with and without A549 cells. For both the untargeted and targeted approach, a compound was only considered to result from alterations in bacterial metabolism due to the presence of alveolar cells when it was a) significantly different from bacteria alone and b) significantly different from cells alone.

## Results

Following data processing and the removal of known analytical artefacts, a total of 503 features were detected and used in the analysis. The impact of alveolar cell presence on microbial derived volatile metabolites was first evaluated using a PCA model based on all 503 detected compounds comparing bacteria and cells cultured in isolation.

### Untargeted analysis

**FIGURE 1:**
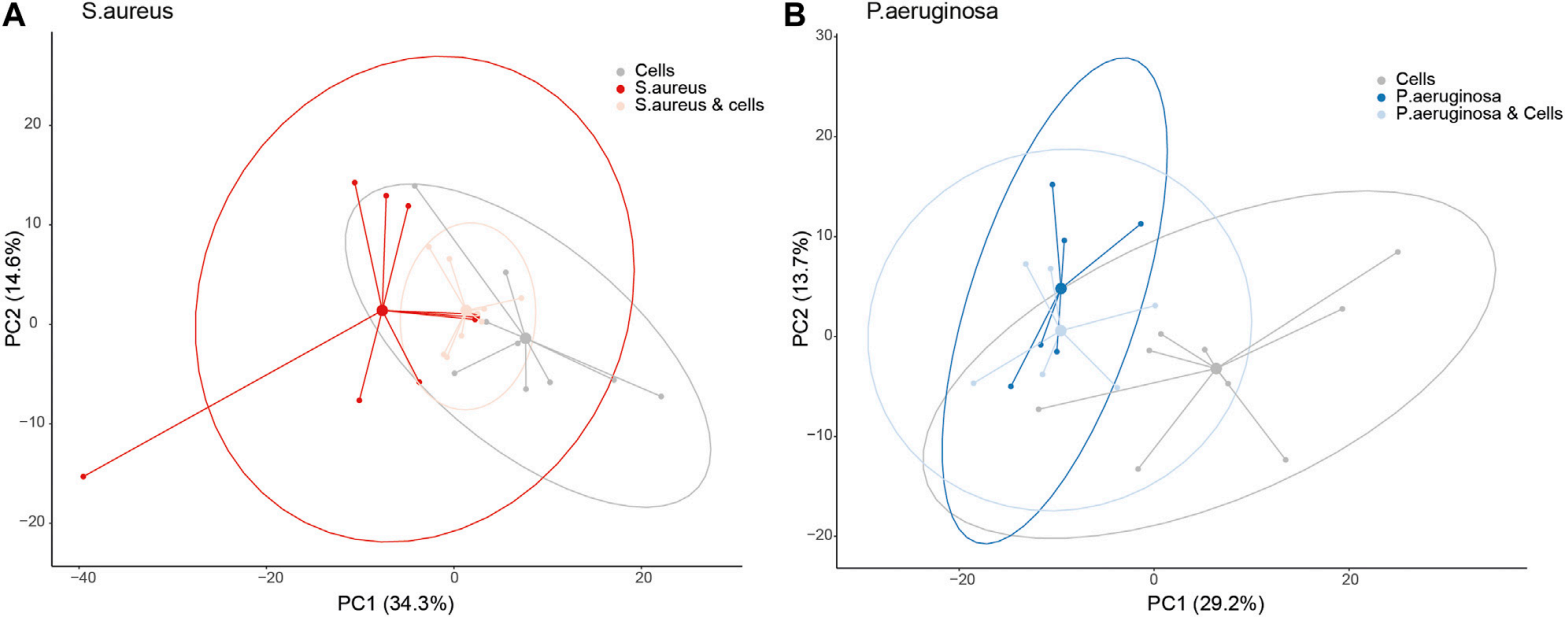
A principal component analysis (PCA) plot for bacteria co-cultured with alveolar cells based on the loadings from a PCA between bacteria and cells in isolation, split per pathogen. Panel **(A)** - *S. aureus* and Panel **(B)** - *P. aeruginosa*.

PCA demonstrated separation between A549 cells *versus S. aureus* or *P. aeruginosa* in culture based on PC1, which explained 34.4% of the total variation for *S. aureus* and 29.2% for *P. aeruginosa* (adjusted *p*-value: 0.0017 and 0.0498, respectively, Figure 1A, B). However, when *S. aureus* was cultured with alveolar cells, the separation from cells in isolation became less clear (adjusted *p*-value: 0.31, Figure 1A). This was not true for the *P. aeruginosa* co-culture where differentiation was still possible from cells in isolation (adjusted *p*-value: 0.028, Figure 1B). The separation between *S. aureus* or *P. aeruginosa* cultured with and without cells was not possible based on the PC1 (adjusted *p*-value: 0.2 and 1.0, respectively).

**FIGURE 2:**
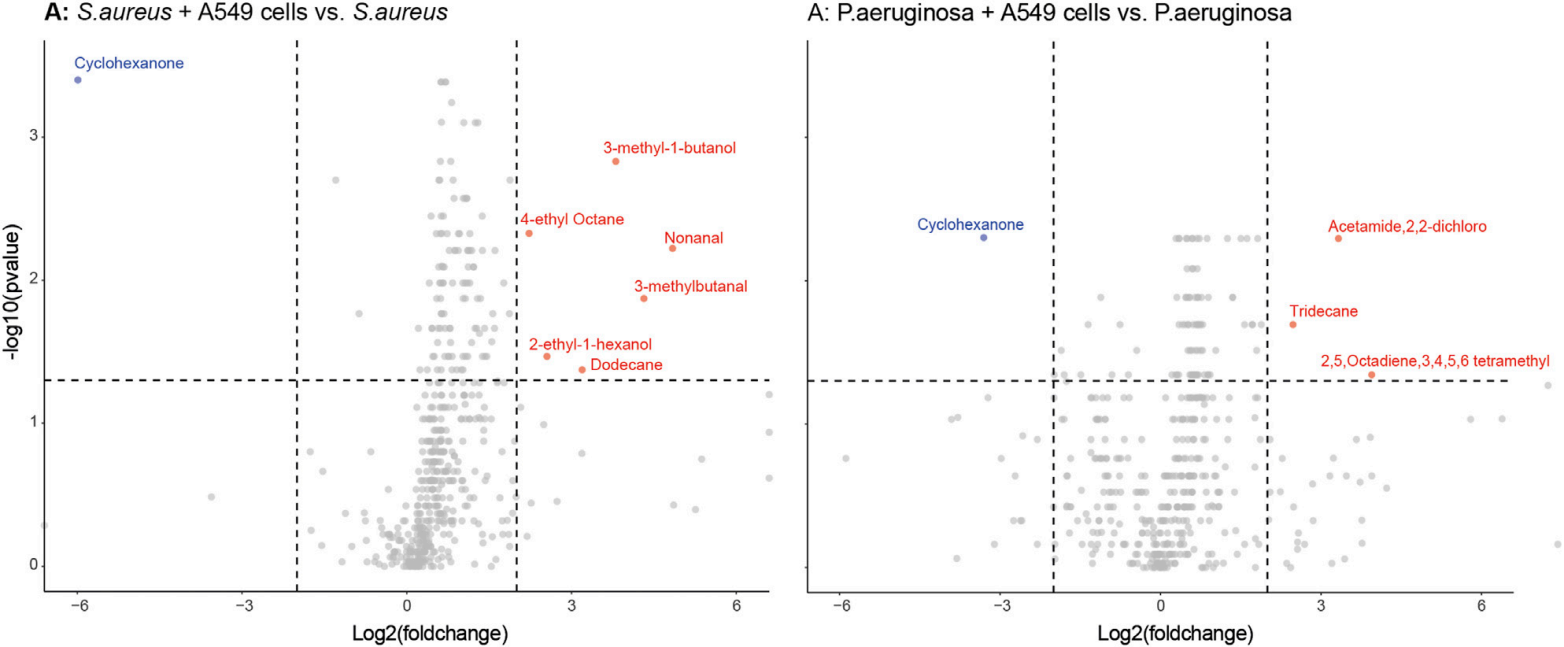
Volcano plots of all VOCs significantly released by either *S. aureus* [Panel **(A)**] or *P. aeruginosa* [Panel **(B)**] when cultured with or without alveolar A549 cells. Blue dots represent down-regulated VOCs and red dots represent up-regulated VOCs with a log2 fold change ≥ 2 and *p* < 0.05 depicted by the dashed line.

**FIGURE 3:**
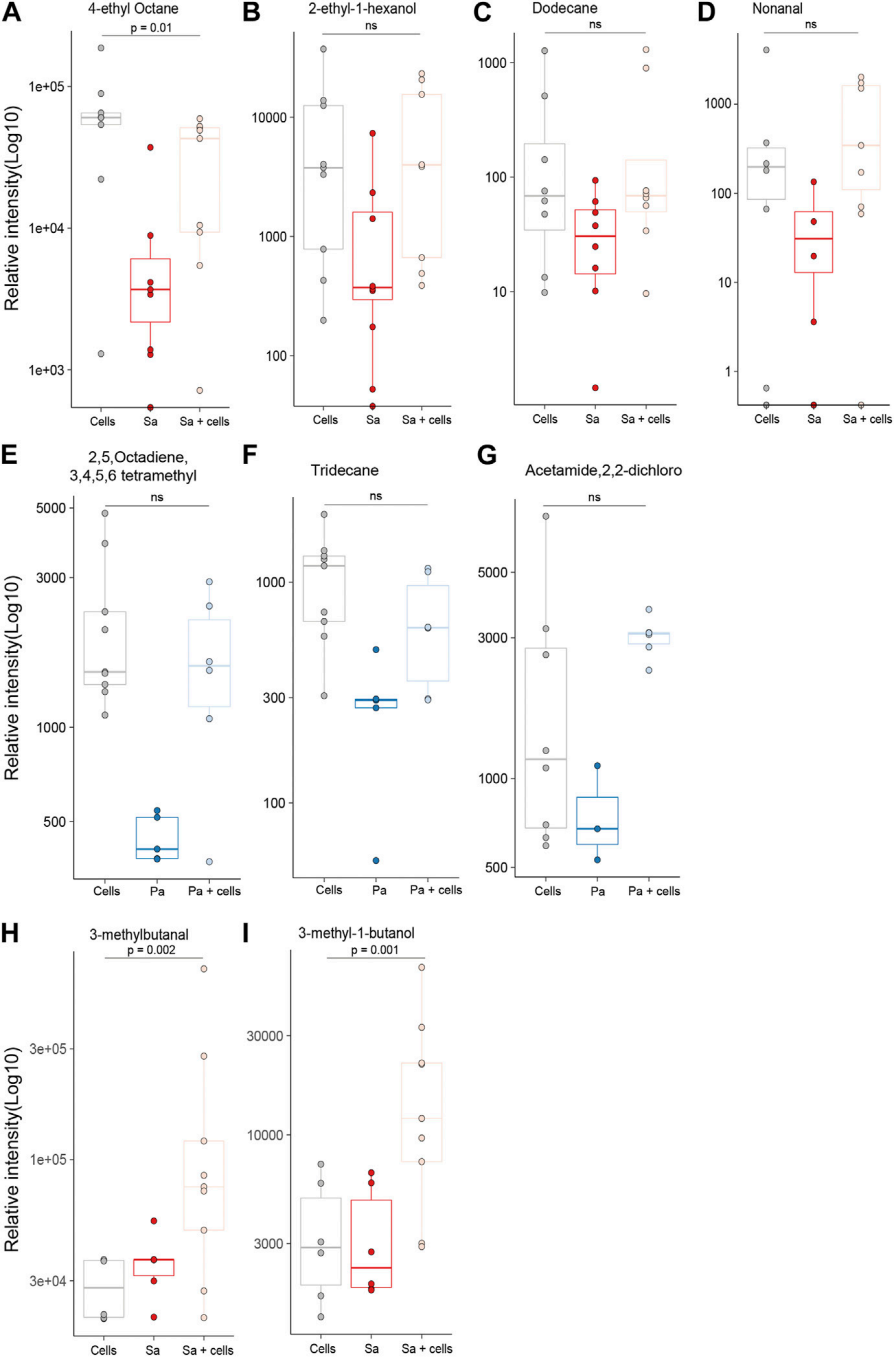
All identified VOCs with a log2 foldchange ≥ 2 and *p* value < 0.05 that showed positive association with bacteria cultured with alveolar cells, split per pathogen. Minimum of six repeats were used to generate the boxplots and *p* value calculated using Mann-Whitney U test. (Cells = A549 cells, Sa = *S. aureus*, Pa = *P. aeruginosa*).

**FIGURE 4:**
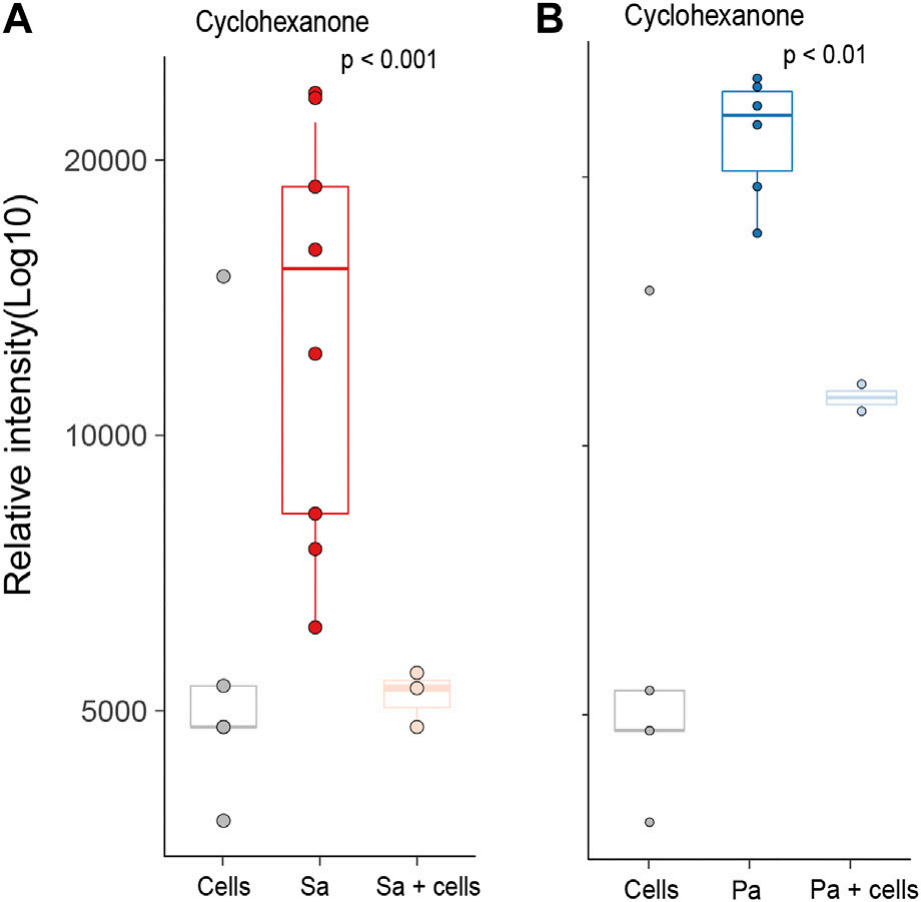
All identified VOCs with a log2 foldchange ≥ 2 and *p* value < 0.05 that showed negative association with bacteria cultured with alveolar cells, split per pathogen. Minimum of six repeats were used to generate the boxplots and *p* value calculated using Mann-Whitney U test. (Cells = A549 cells, Sa = *S. aureus*, Pa = *P. aeruginosa*).

Differences in individual VOCs identified in the headspace of these cultures were further explored through the direct comparison of bacteria cultured with and without alveolar cells, as shown in Figures 2A, B. From the initial 503 detected metabolites, ten (1.9%) compounds met the predefined criteria (log2 fold change ≥2 and *p* < 0.05). Of these, six compounds were found in greater concentration in the *S. aureus* co-culture, three compounds were found in greater concentration in the *P. aeruginosa* co-culture, and one compound was found in lower concentrations in both co-cultures (Figures 2A, B). However, of these nine, six metabolites (2-ethyl-1-hexanol, dodecane, nonanal, 2,5-Octadiene,3,4,5,6-tetramethyl-, tridecane, and acetaminde, 2,2-dichloro) showed no significant difference from alveolar cells alone (Figure 3, B-G, *p* > 0.05). An increased concertation was observed in the cellular headspace for one compound (4-ethyl-octane) compared to the *S. aureus* co-culture (Figure 3A, *p* = 0.01). The two remaining compounds (3-methyl-1-butanol and 3-methylbutanal), found both in higher concentration in *S. aureus* cultured with alveolar cells, were the only metabolites that met the criteria to be considered a result of altered bacterial metabolism due to presence of cells (Figures 3H, I). For both bacteria, cyclohexanone, was found in lower concentration in the co-culture headspace (Figures 4A, B).

### Targeted analysis

Based on previous systematic reviews (Kos et al., 2021; Kos et al., unpublished data), 38 compounds reported in exhaled breath were considered for targeted analysis, including five compounds shared by both *S. aureus* and *P. aeruginosa*, 18 *S. aureus* specific compounds and 15 *P aeruginosa* specific compounds (Supplementary Table S2).

**FIGURE 5:**
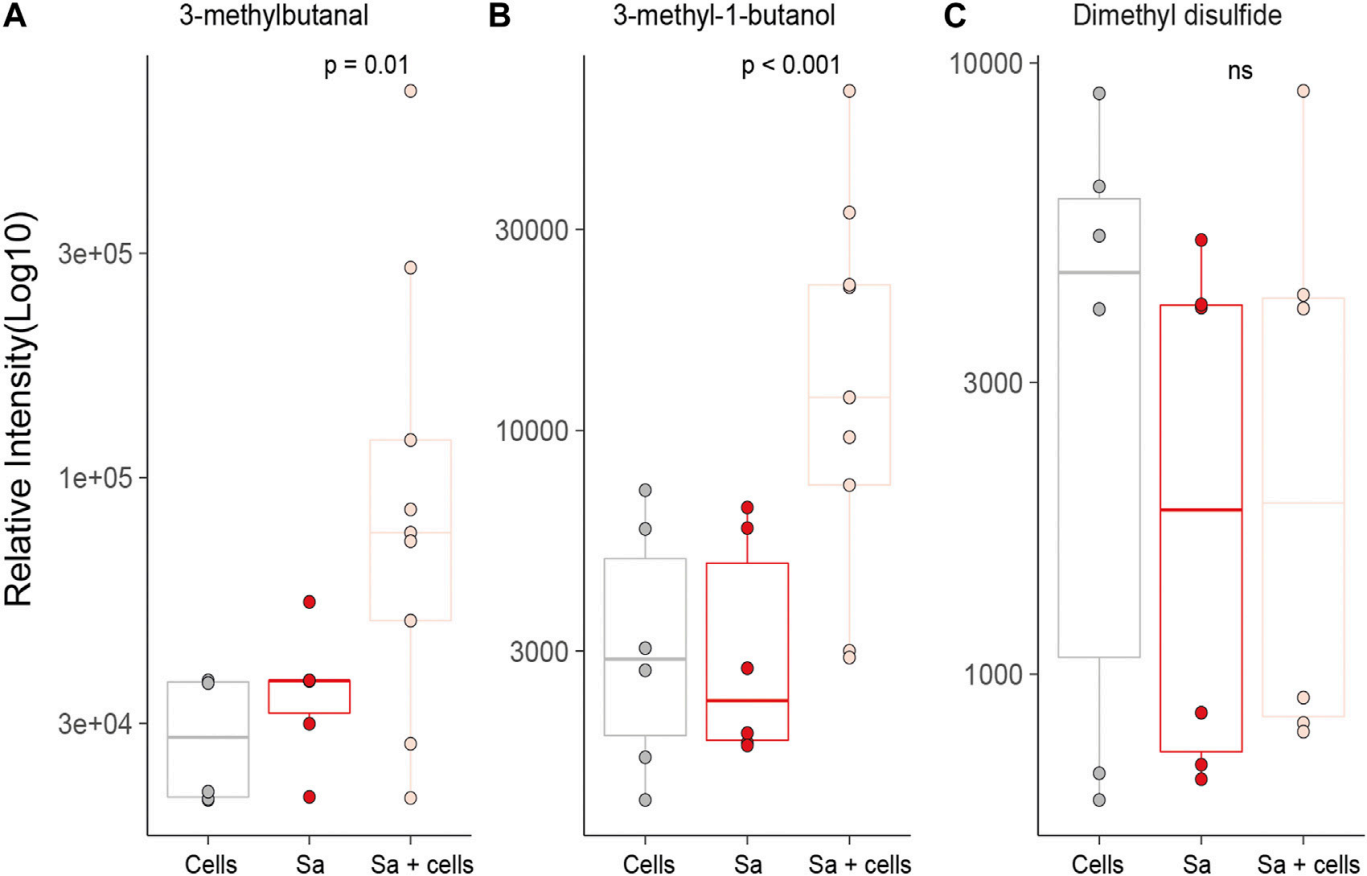
All previously identified target VOCs for *S. aureus* observed in *in vitro* bacterial co-culture headspace, split per treatment group with minimum of six repeats used to create boxplots. Differences between experimental groups evaluated using Mann-Whitney U. (Cells = A549 cells, Sa = *S. aureus*.

Three of the *S. aureus* related molecules were identified from headspaces characterised in this work. One molecule, 3-methylbutanal, was pathogen-specific and two were reported in association with both bacteria (3-methyl-1-butanol and dimethyl disulfide) (Figures 5A–C). Two of three molecules (3-methylbutanal and 3-methyl-1-butanol) showed statistically significant increase in concentration when cultured with alveolar cells compared to alveolar cells in isolation (*p* = 0.001 and *p* = 0.001) or bacteria in isolation (*p* = 0.01 and *p* < 0.001, respectively), see Figures 5A, B.

**FIGURE 6:**
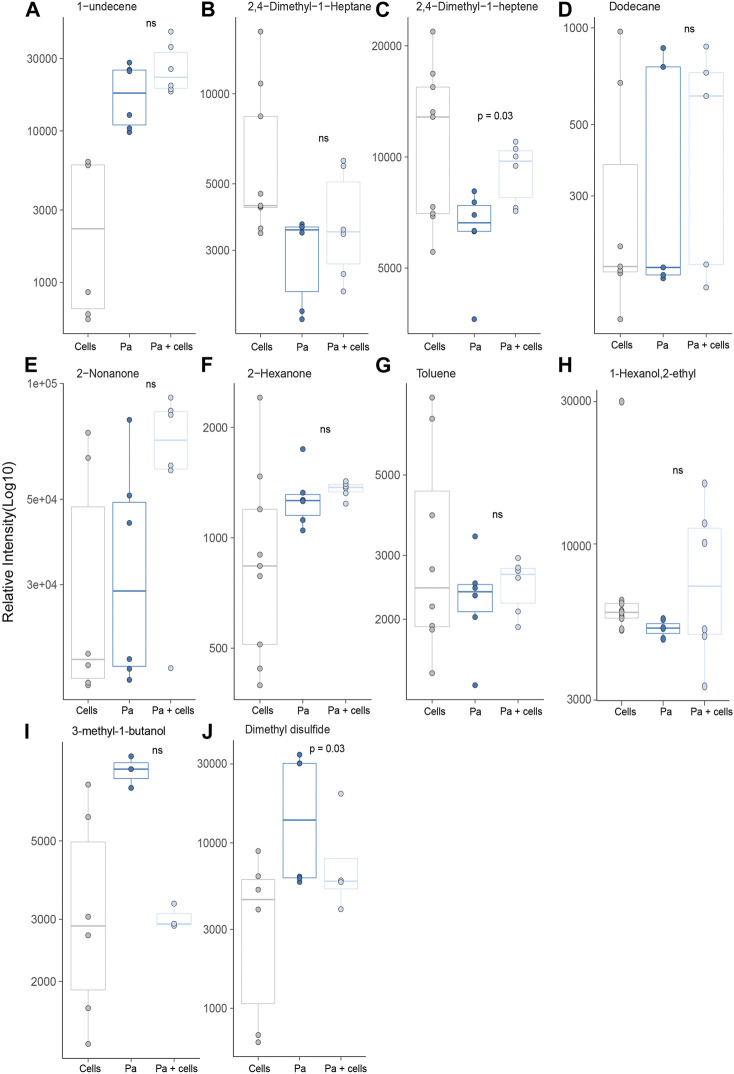
All previously identified target VOCs for *P. aeruginosa* observed in *in vitro* bacterial co-culture headspace, split per treatment group with minimum of six repeats used to create boxplots. Differences between experimental groups evaluated using Mann-Whitney U test. (Cells = A549 cells, Pa = *P. aeruginosa*).

Ten of the *P. aeruginosa* related molecules were identified from headspaces characterised in this work (Figure 6). However, none of these metabolites showed a significant difference when compared to bacteria in isolation and cells alone. In fact, for the majority of the compounds, no differences in headspace concentration were observed between the three experimental groups (Figures 6A–J). One compound, 1-undecene was detected in greater concentration in the bacterial headspace compared to alveolar cells (*p* < 0.001) but its concentration was not altered by co-culture. Two molecules, 3-methyl-1-butanol and dimethyl disulfide, were both found in *S. aureus* and *P. aeruginosa.* However, unlike for *S. aureus*, 3-methyl-1-butanol was not significantly different between the groups (Figures 6I, J). Dimethyl disulfide was found in higher concentrations in the headspace of *P. aeruginosa* cultured in isolation, compared to alveolar cells alone and to co-culture (*p* = 0.01 and *p* = 0.03, respectively, Figure 6J).

## Discussion

HS-GC/MS has been used to provide preliminary data on the influence of clinically relevant nutrients on the production of specific volatile metabolites by *S. aureus* and *P. aeruginosa*, two common respiratory pathogens. Growth of *S. aureus* on alveolar cells rather than culture medium alone resulted in a higher concentrations of 3-methyl-1-butanol and 3-methylbutanal, two important candidate breath biomarkers for *S. aureus* pneumonia.

Growth of *P. aeruginosa* on alveolar cells rather than culture medium alone did not identify statistically significant changes in previously identified VOCs that are associated with pathogens. Together, our results suggest that production of VOCs considered biomarkers of bacterial presence could be influenced by the nutrient environment of the bacterium.

An untargeted approach identified differences in VOC production between bacteria cultured with and without alveolar cells for *S. aureus.* Most noticeably two metabolites, 3-methyl-1-butanol and 3-methylbutanal. These two compounds were also identified as pathogen associated VOC targets having previously been linked to *S. aureus* in numerous studies*,* both *in vitro* (Filipiak et al., 2012; Boots et al., 2014; Chen et al., 2017; Lawal and Muhamadali, 2018; Ahmed et al., 2022) and *in vivo* (Filipiak et al., 2015; Ahmed et al., 2022). However, the differences observed in the current study between *S. aureus* co-culture and bacteria alone may provide further insight into their metabolic origin. Bacterial membrane homeostasis is critical to the survival of *S. aureus in vivo*, ensuring optimal compatibility between the host and pathogen, through the upregulation of fatty acid synthesis (Zhang and Rock, 2008; Parsons and Rock, 2013; Frank et al., 2021). Leucine catabolism is a suggested pathway utilised by *S. aureus* to achieve this (Frank et al., 2021) and leads to the formation of both 3-methyl-1-butanol and 3-methylbutanal as found in the current study. Whilst greater elucidation of the metabolic pathway is required, this study presents additional evidence to support their potential use as biomarkers, in particular, 3-methylbutanal that was recently successfully translated for the identification of *S. aureus* in ventilator-associated pneumonia in a large patient cohort (Ahmed et al., 2022) and warrants further investigation.

Changes to *P. aeruginosa* metabolic activity secondary to host interaction are well documented (Palmer et al., 2007; Jurado-Martín et al., 2021). However, despite this, metabolic changes were not observed in the current study for *P. aeruginosa*. One possible explanation may have been the prolonged incubation period utilised in the current study and the rapid metabolic rearrangement attributed to *P. aeruginosa* virulence (Perinbam et al., 2020). As such, the metabolites reflecting these dynamic changes may have been missed. However, a previous study that similarly co-cultured *P. aeruginosa* with alveolar cells also showed no differences between bacteria with and without cells despite a shortened incubation window (Lawal and Knobel, 2018). Alternatively, it has been postulated that pseudomonal adaptation and successful colonisation are dependent on the nutritional components found in sputum (Palmer et al., 2007). As such, it is more likely that the co-culture model presented here may have provided inadequate nutritional cues to stimulate meaningful metabolic re-arrangement despite using nutrients more representative of *in vivo* conditions, reinforcing the importance of carefully defining infection site physiology for future studies and considering not only anatomical location but also pathogen prerequisites.

In addition to the untargeted analysis, we also performed targeted analysis in which previously identified microbial VOCS were sought to evaluate if the use of a more physiologically relevant growth media might lead to an increased headspace concentration. Whilst this was true for *S. aureus* co-cultures for both 3-methyl-1-butanol and 3-methylbutanal, the same cannot be said for *P. aeruginosa,* where differences in VOC release could not be attributed to changes in its metabolism resulting from co-culture with alveolar cells. Furthermore, most of the previously identified *P. aeruginosa* VOC biomarkers showed no significant difference in headspace concentration of *P. aeruginosa* when compared to alveolar cells alone. This differs from the results found in the systematic review conducted by Kos et al., 2021, that identified a number of *P. aeruginosa* specific VOCs. It is important to recognise however, that in the majority of these studies bacteria were cultured in isolation and compared to other bacterial species, not human cells lines. Subsequently, for these studies, VOC release may have been attributed to the bacteria, whereas the current study would suggest their origin is not necessarily only microbially derived and instead could reflect metabolic changes secondary to their pathogenicity. This is particularly evident in the case of dimethyl disulfide, which returned to alveolar cell concentrations when co-cultured with them. As such, the clinical utility of these biomarkers is questionable and caution is warranted for their use *in vivo*. This was not true for all targeted VOCs. 1-Undecene did show a positive association with *P. aeruginosa* greater than alveolar cells in isolation and is in agreement with numerous other studies as evidenced by Kos et al., 2021. However despite this, its translation from pre-clinical to clinical studies is yet to be proven (Kos et al., 2021; Ahmed et al., 2022) and suggests that further work is needed to better determine its *in vivo* biosynthesis before its use as VOC biomarker can be recommended.

A strength of this study lies in the use of a previously developed headspace model that incorporates both glass culture vessels and a sealed system to minimize the impact of plastic contaminants and improve reproducibility (Ahmed et al., 2022; Fenn et al., 2022). Furthermore, the incorporation of bacterial co-culture demonstrated the versatility of said model and could provide an easy and reliable platform to further examine the nutritional effects of more *in vivo* relevant growth media on VOC production. Additionally, the approach taken represents the culmination of comprehensive systematic reviews coupled with a validated *in vitro* model for a reliable VOC assessment utilising both targeted and untargeted analyses.

The key limitation of this approach was the use of a two-dimensional culture model over more physiological relevant three-dimensional models, such as air liquid interface cultures. This model was used however to maximise VOC capture and minimise the influence of VOCs omitted by plastic equipment used in such models. The low number of bacterial species and use of a single cell line is another limitation that warrants caution when extrapolating the current findings to other bacteria or infection sites. Further work therefore should include additional bacterial species and strains co-cultured with different cell types to validate the presented findings. Finally, the respiratory tract is a complex and dynamic community of microbiota (Dickson et al., 2015). As such, the use of bacterial monocultures in the current work is another limitation that should be considered when interpreting our results. The approach taken here likely underestimates *in vivo* bacterial pathogenesis and fails to capture all the interactions between pathogenic bacteria and other respiratory tract flora that could influence VOC production. However, the integration of multiple bacterial interactions represents a challenge when attempting to trace the VOC origin. A challenge faced by *in vivo* exhaled breath analyses and the models that investigate them, such as presented in this study, may be in a better position to address these challenges. Nevertheless, we recognise this as a limitation and advise that caution is taken when interpreting the results in a clinical environment.

Overall, the detection of pathogen associated compounds was poor for both bacteria. Target compounds were either not detected, or showed no significant difference between experimental groups. Similar difficulties have also been observed in other studies attempting to translate volatile metabolites (Filipiak et al., 2015; Kos et al., 2021; Ahmed et al., 2022). However, more often than not, the emphasis is primarily given to the compounds that are found, with the translational discrepancies either not being discussed or attributed to differences in sorbent materials, column affinities or analytical methodologies. Whilst we recognise the validity of such arguments, they represent an innate problem in exhaled breath research that continues to complicate its clinical integration. This burgeoning challenge is further exacerbated by the continued development of new technologies, lack of consensus on which to use and penchants for discovery studies. As such, a plethora of VOC biomarkers continue to be identified that may in fact represent the techniques used rather than their applicability in the pathologic condition studied. Therefore, if exhaled breath is to become clinically applicable, the preference for discovery studies must be disrupted and the emphasis given to a) translational and development studies b) the development of better *in vitro* models c) the use of multiple analytical techniques and d) the cross validation with external research institutions.

## Conclusion

HS-GC/MS analyses showed that nutrients more representative of *in vivo* environment had an influence on the production of specific volatile metabolites by *S. aureus* and *P. aeruginosa.* The observed differences for *S. aureus* identified two important candidate biomarkers, 3-methyl-1-butanol and 3-methylbutanal. The fewer discernible differences for *P. aeruginosa* challenges the validity of previously suggested breath biomarkers. Together, our results suggest that VOC biomarkers may indicate the presence of bacterial species, and are influenced by the local nutritional environment and should be considered when evaluating their biochemical origin.

## Data Availability

The raw data supporting the conclusion of this article will be made available by the authors, without undue reservation.
