# Supplementary material for: Influence of bacterial and alveolar cell co-culture on microbial VOC production using HS-GC/MS
Source: Front Mol Biosci. 2023 Apr 26;10:1160106. doi: 10.3389/fmolb.2023.1160106 (PMC10169821; doi:10.3389/fmolb.2023.1160106)
Supplement: Supplementary file 1 [file Table1.DOCX]

**Supplementary material**

**Table S1:**

List of previously reported in vivo VOCs associated with either *S.aureus* or *P.aeruginosa*. Modified list taken from work done by (Kos *et al.*, 2021, Kos *et al*., unpublished data)

|  | **Compound** | **CAS. No** | ***S.aureus*** | ***P.aeruginosa*** |  |
| --- | --- | --- | --- | --- | --- |
| Hydrocarbons | Butane | 106-97-8 | + |  |  |
|  | 1,3−butadiene | 106-99-0 | + |  |  |
|  | Propane | 74-98-6 | + |  |  |
|  | 2-methylpropene | 115-11-7 | + |  |  |
|  | 1−undecene | 821-95-4 |  | + |  |
|  | 2,4−dimethyl−1−heptane | 2213-23-2 |  | + |  |
|  | 2,4-dimethyl-1-heptene | 19549-87-2 |  | + |  |
|  | Dodecane | 112-40-3 |  | + |  |
|  | Isoprene | 78-79-5 |  | + |  |
| Alcholols | 2-ethylhexanol | 104-76-7 |  | + |  |
|  | 1-Propanol, 2-methyl | 78-83-1 | + |  |  |
|  | Ethanol | 64-17-5 | ++ | ++ |  |
|  | 3-methyl-1-butanol | 123-51-3 | + | + |  |
| Acids | 3-methylbutanoic acid | 503-74-2 | ++ |  |  |
|  | Acetic acid | 64-19-7 | + |  |  |
| Ketones | 2−pentanone | 107-87-9 | + | + |  |
|  | Acetol (hydroxyacetone) | 116-09-6 | + |  |  |
|  | Acetoin | 513-86-0 | ++ |  |  |
|  | Acetone | 67-64-1 | + | + |  |
|  | 2−butanone | 78-93-3 | + |  |  |
|  | 2-nonanone | 821-55-6 |  | + |  |
|  | 2−hexanone | 591-78-6 |  | + |  |
|  | 2−heptanone | 110-43-0 |  | + |  |
| S-containing | DMDS | 624-92-0 | + | + |  |
|  | Methanethiol | 74-93-1 | + |  |  |
| Aldehydes | Propanal | 123-38-6 | + |  |  |
|  | 3-methylbutanal | 590-86-3 | ++ |  |  |
|  | Methacrolein | 78-85-3 | + |  |  |
| Esters | Ethyl isovalerate | 108-64-5 | + |  |  |
|  | Ethyl acetate | 141-78-6 | + |  |  |
|  | Isoamyl acetate | 123-92-2 | ++ |  |  |
| Alkenes | (Z)-2-butene | 590–18–1 | + |  |  |
| Cyclics | Limonene | 138-86-3 |  | ++ |  |
|  | Toluene | 108-88-3 |  | + |  |
| N-containing | 2−aminoacetophenone | 551-93-9 |  | ++ |  |
|  | Acetonitrile | 75-05-8 |  | + |  |
|  | Ammonia | 7664-41-7 |  | + |  |
|  | Hydrogen−cyanide | 74-90-8 |  | ++ |  |

*“+” indicates the production of a VOC by a pathogen, “++” indicates VOC reported ≥ 2 in literature*

**References**

Kos, R. *et al.* (2021) ‘Targeted exhaled breath analysis for detection of Pseudomonas aeruginosa in cystic fibrosis patients’, *Journal of Cystic Fibrosis*. Elsevier B.V., (xxxx). doi: 10.1016/j.jcf.2021.04.015.
